# High-resolution co-seismic fault offsets of the 2023 Türkiye earthquake ruptures using satellite imagery

**DOI:** 10.1038/s41598-024-55009-5

**Published:** 2024-03-21

**Authors:** Floriane Provost, Volkan Karabacak, Jean-Philippe Malet, Jérôme Van der Woerd, Mustapha Meghraoui, Frédéric Masson, Matthieu Ferry, David Michéa, Elisabeth Pointal

**Affiliations:** 1 Ecole et Observatoire des Sciences de la Terre (EOST), CNRS UAR 830, Université de Strasbourg, 5 rue René Descartes, 67084 Strasbourg, France; 2 Institut Terre et Environnement de Strasbourg (ITES), CNRS UMR 7063, Université de Strasbourg, 5 rue René Descartes, 67084 Strasbourg, France; 3 Department of Geological Engineering, Eskisehir Osmangazi University, 26040 Eskisehir, Turkey; 4 Géosciences Montpellier, CNRS UMR 5243, Université de Montpellier, Montpellier, France; 5 Data-Terra / Pôle de Données Terre Solide (ForM@Ter), CNRS, Institut de Physique du Globe de Paris, 1 rue Jussieu, 75005 Paris, France

**Keywords:** Tectonics, Natural hazards, Tectonics, Natural hazards

## Abstract

On February 6, 2023, southern Türkiye was struck by two large earthquakes at 01:17 UTC (Mw=7.8, Pazarcık, Kahramanmaraş) and 10:30 UTC (Mw = 7.6, Elbistan, Kahramanmaraş), causing severe damage at the complex junction of the Dead Sea Fault (DSF), the Cyprus Arc and the East Anatolian Fault Zone (EAFZ). The ruptures propagated along several known strands of the southwestern termination of the EAFZ, the main Pazarcık and Karasu valley faults, and the Çardak-Sürgü fault. Here we present the high-resolution mapping of the entire coseismic surface rupture and an estimate of the rupture width, total and on-fault offset, and diffuse deformation obtained a few days to three months after the two mainshocks. The mapping is derived from image correlation of Sentinel-2 optical satellite imagery and validated with offset measurements collected on the ground. We find that the ruptures extend over lengths of 310 km and 140 km for the Mw 7.8 and Mw 7.6 mainshocks, respectively. The maximum offsets reach 7.5 ± 0.8 m and 8.7 ± 0.8 m near the epicenters of the Mw 7.8 and Mw 7.6 events, respectively. We propose a segmentation of the two ruptures based on these observations, and further discuss the location of the potential supershear rupture. The use of optical image correlation, complemented by field investigations along earthquake faults, provides new insights into seismic hazard assessment.

## Introduction

**Figure 1:**
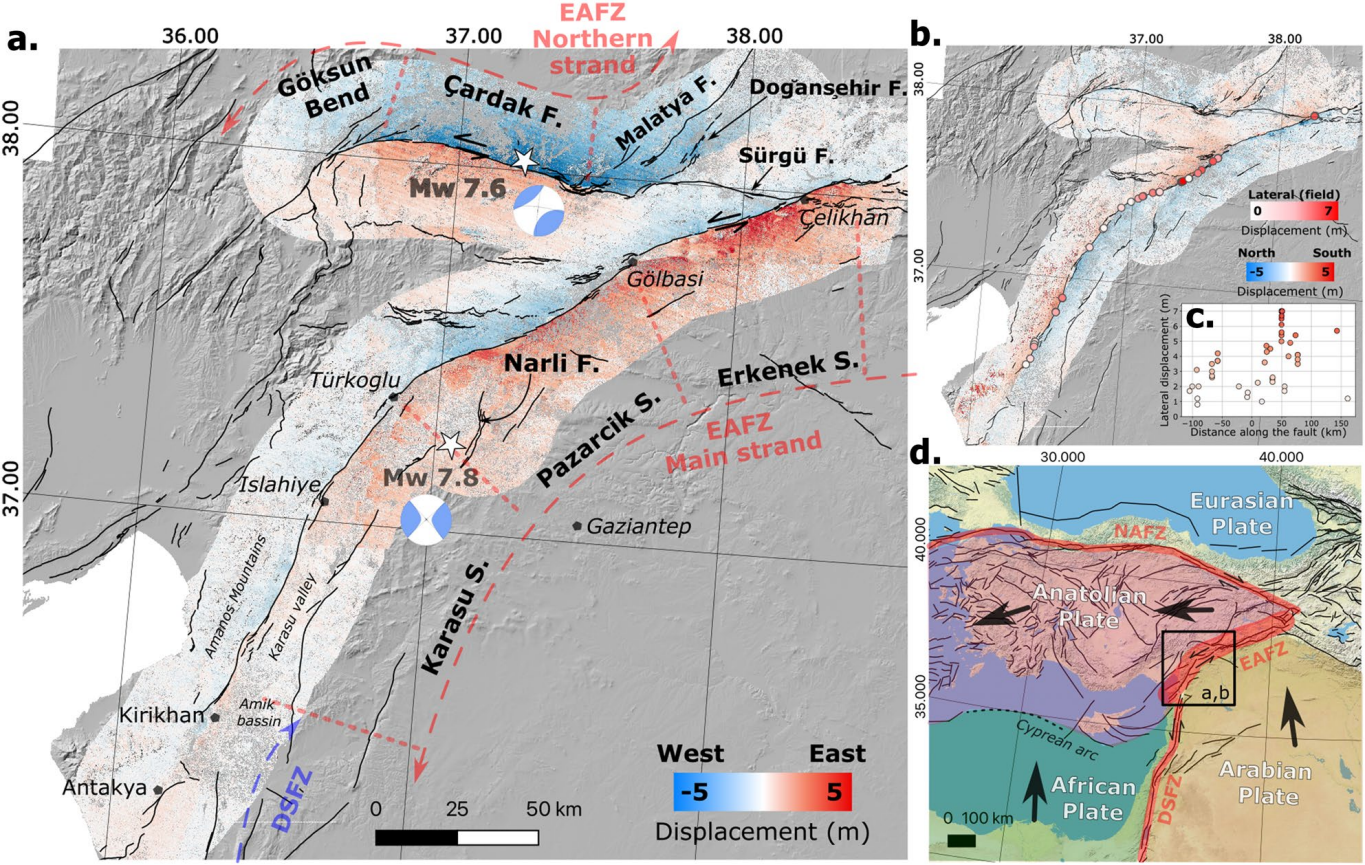
Co-seismic displacements for the 2023 Kahramanmaraş doublet. The Mw 7.6 and Mw 7.8 epicenters are represented with white stars and their associated focal mechanisms^31^. East-West and North-South displacement fields computed with the GDM-OPT-ETQ processing chain applied to Sentinel-2 optical satellite imagery are presented in (**a,b**). (**a**) Displays the regional context of the southern part of the EAFZ: the black lines show the database of active faults in the region^15^ and the segments of the main and northern strands of the EAFZ are indicated^14^. (**b**) Displays the 48 field measurements (circles) taken in May 2023. The measured offsets along the main fault line are shown in (**c**), and the regional tectonic context is presented in (**d**) (active fault of^32^.

The East Anatolian Fault Zone (EAFZ) has been the site of several large earthquakes in the past^1^ and accommodates the left-lateral movement between the Arabian and Anatolian plates (Fig. 1d). The historical seismicity catalogue revealed a seismic quiescence of the SW segments with respect to the NE section of the EAFZ^2^. A recent Global Navigation Satellite Systems (GNSS) study derived a slip rate of 9.2±0.5 mm.year^-1^ on the Pazarcık and Erkenek segments^3^ (Fig. 1a) with an estimated strain accumulation of about 8 metres since the 1114 AD earthquake (Ms > 7.8)^3^. Therefore, earthquakes of magnitude Mw 7.2–7.6 have been hypothesised to occur in this part of the EAFZ^4^. In the southern part of the EAFZ on the Karasu segment (Fig. 1a), a lower slip rate of 4.5±1.1 mm.year^-1^ is determined^3,5^. Large historical earthquakes occurred along this segment in 1822 A.D. (Ms 7.0) and 1872 A.D. (Ms 7.2), resulting in strain accumulation of 0.6–1.1 m^3^. Potentially, earthquakes of Mw 6.8-7.2 were expected in the future in this segment of the EAFZ^4^.

On February 6, 2023, two earthquakes struck the region, causing at least 65,000 deaths and widespread damage in Türkiye and Syria. A first mainshock of Mw 7.8 was followed 8 hours later by a Mw 7.6 earthquake. The Mw 7.8 rupture started on the Narlı fault, 15 km south of the EAF main strand (Fig. 3a) and later propagated bilaterally along the EAF main strand along the Erkenek segment to the northeast and along the Pazarcık and Karasu/Amanos segments to the southwest^6–11^. The second earthquake started on the Çardak fault (Fig. 1a) and propagated bilaterally west and east towards the east and west extremities of the Çardak fault (Fig. 3a). The 2023 doublet is the strongest earthquake documented on the EAFZ since 1114 CE^6,10^ and provides a unique opportunity to map the active faults in the region.

Indeed, several studies have suggested various segmentation models of the EAF that differ on the location of the active faults and active plate margins in this region^3,12–16^. The debate concentrates in the south-western part of the EAFZ from Çelikhan to the south of Antakya where it connects with the Dead Sea Fault Zone (DSFZ) (Fig. 1a). In fact, the connection between these two major fault zones remains uncertain. Among the different models, some hypothesized that the EAFZ continues from Türkoğlu westwards to the Cyprus arc/subduction, over the north of the Amanos mountain range and along the Karasu valley towards the DSFZ with discontinuous faulting^3,17–20^. Other models proposed that the main EAF strand continues south from Turkoglü along the Karasu valley and the eastern side of the Amanos mountain range^12,14^ to reach the junction with the DSFZ in the Amik basin. Finally, the existence of a northern strand along the Çardak-Sürgü fault belonging to the EAFZ has been proposed^14,15^. The same authors^14,15^ define the main strand as a continuous fault system along 580 km from Karlıova in the northeast (i.e. junction point with the North Anatolian Fault) to Antakya in the southwest (Fig. 1a). The precise mapping of the rupture of the 2023 earthquake sequence is here critical for the understanding of the coseismic rupture and strain distribution at the triple plate boundary between Arabia, Anatolia, and Africa.

Satellite imagery has proven to be an important source of information to map the surface rupture and its structural complexity, and to determine co-seismic offsets and diffuse rupture zone widths^21–26^. The current availability of Earth Observation satellites with regular acquisitions and global coverage now provides rapid post-event acquisitions that can be integrated into models with short delays^7,11,27–30^. However, these models usually use a simplified trace of the rupture and may neglect near-fault displacements, leading to partial conclusions. This paper presents a complete estimation of the surface ruptures and their geometric properties (e.g. fault zone dimensions, diffuse deformation) from high-resolution offset measurements.

## Co-seismic displacement of the Kahramanmaraş doublet derived from pairwise image correlation

Synthetic Aperture Radar satellites (SAR) such as Sentinel-1 or ALOS-2 can provide the full 3D displacement maps but with a spatial resolution of 30–60 m^33^. In the case of the Kahramanmaraş doublet, the vertical motion from the inversion of the 3D displacement fields appears to be small (< 1.5 m^28,33,34^) and very localized compared to the horizontal displacement fields (> 3 m;^28,33,34^). In this study, we therefore choose to work only with Sentinel-2 images at full resolution (i.e. 10 m $\times$ 10 m) in order to derive 2D co-seismic displacement fields and rupture location with a better resolution. The co-seismic displacements are computed using the GDM-OPT-ETQ processing chain^35^ based on the MicMac image matching library^36,37^ (see “Methods” for more details). Two pairs of images are correlated and spatially averaged (Fig. 1a, b). The first computed pair covers the narrowest possible temporal baseline with cloud-free Sentinel-2 images of January 25, 2023, and post-earthquakes images of February 9, 2023 (Fig. S1a). The displacements measured with this pair are highly contaminated with topographic noise due to the presence of snow in the northern part of the region (Fig. S1a). To reduce this noise, a second pair is computed (Fig. S1b), with a temporal baseline of one year, using the images of May 20, 2022, and May 25, 2023 (i.e. the first cloud-free and snow-free acquisitions after the earthquakes). In total, 9 Sentinel-2 tiles of 100 $\times$ 100 km were processed (Table S1) for a total of 300 $\times$ 300 km of mapped co-seismic displacements, which shows the exceptional spatial extent of the rupture of the 2023 Kahramanmaraş doublet (Fig. 1a, b).

**Figure 2:**
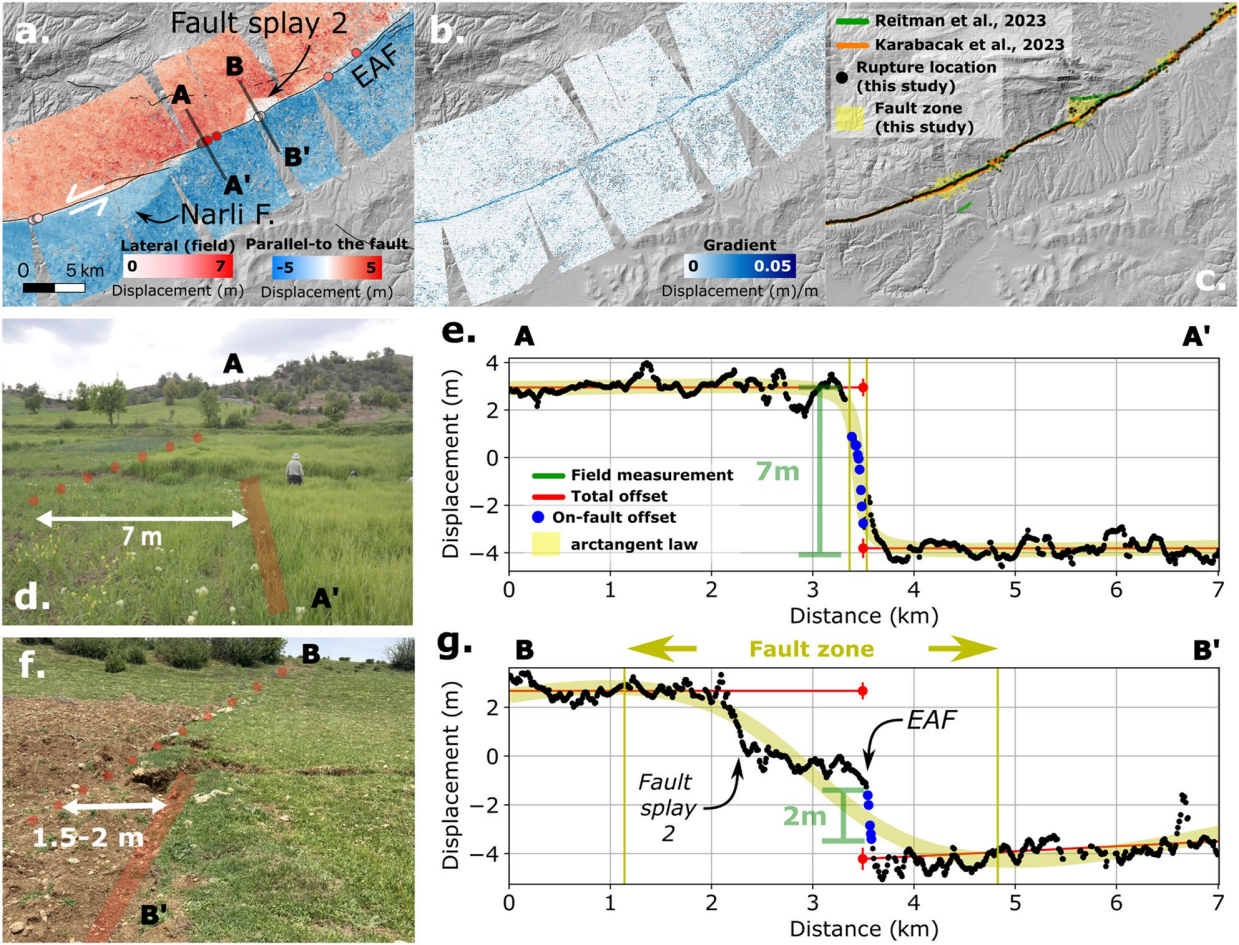
Co-seismic rupture traces derived from co-seismic satellite displacements fields and field observations: (**a**) example of parallel to the fault displacements (computed along the direction of the EAF) at the junction of the Narli fault and the EAF; (**b**) gradient of the parallel to the fault displacements computed along profiles perpendicular to the direction of the EAF; (**c**) rupture traces (in black dots) derived from the gradient together with the rupture traces mapped by other authors^6,39^. The total fault zone width derived from the arctangent law (see Methods for more details) is shown in yellow; (**d**,**f**) are field pictures of the offset measured on the field at the intersection between the AA’ and BB’ profiles (Fig. 2a); (**e,g**) are examples of parallel-to-the fault displacement measurements from satellite image correlation (black dots) along the profiles AA’ and BB’. The measured rupture properties (i.e. total offset, on-fault offset and fault width) and the field measurements are represented together.

**Figure 3:**
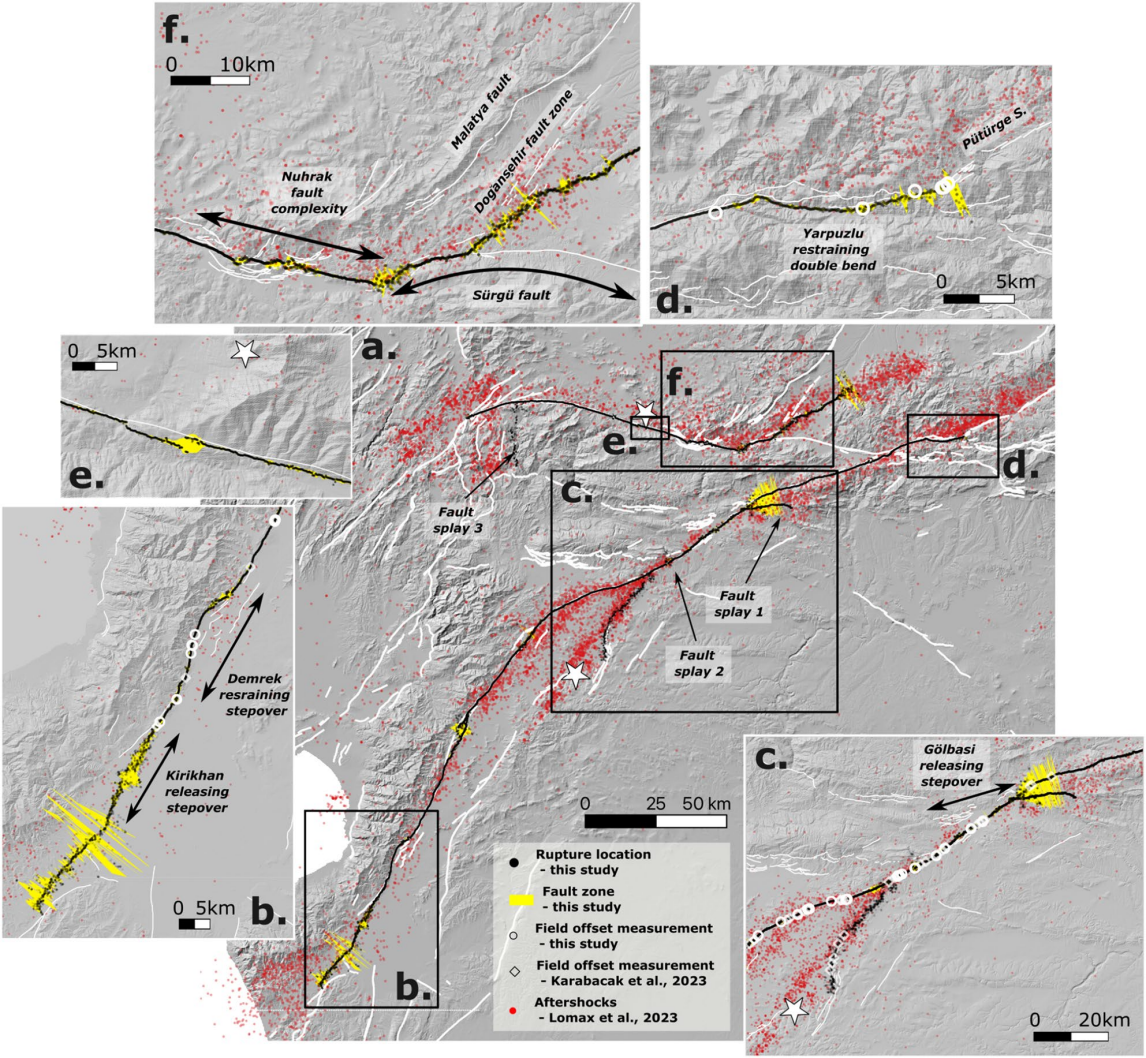
Surface rupture triggered by the Kahramanmaraş 2023 doublet: (**a**) surface rupture for the Mw 7.6 and Mw 7.8 earthquakes (black dots) plotted together with the aftershock locations from^31^ (red dots). The epicenters of the main shocks relocated by^31^ are represented by white stars. White lines represent the database of active faults^15^ and the yellow area represents the total fault width derived from satellite image correlation (see “Methods” for details). (**b**–**f**) Zooms of several zones as well as the offsets measured in the field (white diamonds and circles): (**b**) shows a zoom of Narli and the EAF junction near the Mw 7. 8 epicenters; (**c**) shows a zoom on the previously unmapped fault trace; (**d**) shows a zoom on the previously unmapped fault trace to the west of the Golbasi releasing bend; (**e**) shows a zoom on the fault trace near the Mw 7.6 second main shock; (**f**) shows a zoom on the Demrek restraining bend. The background of the maps is the hillshade of the 2021 GLO30 Copernicus DEM at 30m spatial resolution, https://doi.org/10.5270/ESA-c5d3d65.

The East–West (EW) displacements reach an absolute magnitude of 5 m along a large portion of the Erkenek and Pazarcık segments of the EAFZ and along the Çardak fault (Fig. 1a) while, in the North-South (NS) direction, the displacements are much smaller, reaching 3 m and are more localized (Fig. 1b). The satellite observations are further used to quantify the displacement parallel- and perpendicular to the fault trace (Fig. 2a, Figs. S2–S5) along more than 40,000 profiles spaced every 10 m along the fault direction and locally perpendicular to it. The gradient is calculated along each profile (Fig. 2b) and the rupture is determined based on a threshold of 0.02 (m/m) (Fig. 2c, see Methods for more details). The automatic location of the rupture allows the fault to be mapped in detail (Figs.  2c,  3) along the entire length of the rupture where offsets are greater than 1 meter (i.e. 1/10th of the pixel size, the precision of image correlation^38^). We compare the result with the location of the rupture made in the field (Figs. 1b,  2d,  f) and from^6^ for a total of 108 points along the Mw 7.8 rupture. On average, the distance between the satellite-derived rupture location and the field-observed location is 28 m, with a minimum of 2.5 m and a maximum of 143 m. Finally, the rupture width, the total and on-fault offset are computed (Fig.  2e, g) every 5 cross-profiles (50 m) along all mapped faults (see “Methods” for more details).

## Active faults of the Kahramanmaraş doublet

Overall, we map a 310 km-long rupture with a left-lateral NNE trend associated with the first Mw7.8 mainshock and comprising the Karasu, Pazarçık and Erkenek segments (Fig. 3a). In the southern part of the Karasu segment, the rupture trace can be traced to the DSFZ with a large releasing stepover south of Kirikhan (Fig. 3a, b), where liquefaction was observed^6,40^. The extremity of the EAFZ was previously poorly known and terminated south of Kirikhan with a slight deviation towards the ENE^14,15^. The trace mapped in this study suggests that the EAFZ extends 30 km further south to Antakya with a NNE trend (Fig. 3) that connects to the Antakya fault zone^14,15^. The closest aftershocks are spreading along the Amanos Mountains in an ESE direction to the north of the mapped trace (Fig. 3a). These locations can be explained by the occurrence of a Mw 6.4 event on February 20, 2023, which activated another fault^7^.

At the northeastern extremity of the Mw 7.8 rupture, the rupture trace is clearly visible up to the end of the Yapurzlu releasing bend, and corresponds to the end of the rupture mapped in the field (Fig. 3d) where the trace on the ground gradually disappears. Numerous aftershocks occurred in the first weeks after the mainshocks over a distance of 30 km on the Pütürge segment (Fig.  3a), leading to several interpretations. Jia et al. (2023)^11^ suggest in their model that a sub-event was initiated at the Yapurzlu complex and propagated along the Pütürge segment with a slip of 1 to 2 m in the shallowest part of the fault, while in the model proposed by Barbot et al.(2023)^7^, the rupture is stopped at the Yapurzlu complex, raising concern about the presence of a seismic gap on the Pütürge segment. In the case of satellite image correlation, the detectability of surface displacements is strongly dependent on the spatial resolution of the images. The Sentinel-2 derived displacement fields, presented here, are limited and are accurate for displacements larger than 1-1.5 meter. However, there is a slight offset visible along the Pütürge segment over about over 20 km (Fig. S6), consistent with the direction of the aftershocks (Fig. 3a), suggesting that the rupture has propagated along the Pütürge segment.

It is noteworthy that the Mw7.8 mainshock originated on the Narli fault where the rupture is visible to the north of the previously mapped trace^15^ (Fig. 1b). The trace of the mapped rupture is consistent with the data collected in the field^6^ (Fig. 3c), while the epicenter is located further to the southwest approximately 15 km from the southern end of the mapped fault trace (Fig. 3c). The direction of the mapped fault trace differs from the direction of the aftershocks in the south (Fig. 3a, c), suggesting that the fault may either be dipping to the west, which would be consistent with the interpretation of the normal mechanism proposed by previous studies^14,15^, or might splay. To the north, the Narli fault continues to the EAF in the region where maximal offsets are measured (Fig. 3c). The location of the junction seems to vary between studies, depending on the data analyzed. Similar to^30^, who analyzed Sentinel-2 data, we propose to locate the junction a few kilometers more to the east (i.e. 37.56784 N, 37.29324 E; Figs. 2a, 3c) than suggested by other studies^7,11,29,39^ who analyzed Sentinel-1 or ALOS-2 data. This is supported by the observation of a slight offset (< 2 m) in the North-South component and by the distribution of the aftershocks (Fig. 3c).

On the Çardak fault, where the Mw7.6 rupture was initiated and propagated, a 140 km long rupture with a left-lateral east-north-east (ENE) strike is visible (Fig. 3a). It follows the Çardak fault to the Göksun bend to the west and propagates to the east in the vicinity of the Doğansşehir fault zone, south of the Malatya fault (Fig. 3f). The Nurhak fault complexity is clearly observed^14^, but surprisingly, further to the east, the rupture propagates a few kilometers along the Sürgü fault and, then, instead of propagating along the entire Sürgü fault to join the EAF, the rupture continues in the NE direction along an unmapped fault south of the Doğansşehir fault zone (Fig. 3f). The northeastern end of the rupture is difficult to determine because of the very small displacements (< 1.5 m; Fig. S6), which are below the sensitivity of image correlation with Sentinel-2 data. The distribution of the aftershocks suggests that the rupture has propagated 17 km further to the NE (Fig. 3, Fig. S6) compared to the rupture mapped in this study. We therefore delineate three main segment with the Göksun bend, the Çardak fault and the Nur.

In addition, two fault splays can be mapped in the Pazarcık segment (Fig. 3a), that were not previously documented^14,15,41^. The first one, located on the east of the Gölbasi releasing stepover, is a 12.5 km long fault splay trending to the East (Fig. 3a, c). A second west-trending 2 km-long splay is visible to the North of the Narli and EAF junction (Figs. 2a–c, 3a). To the south of the Göksun bend, a south-striking, 20 km-long fault splay is also detected (Fig. 3a). This fault is interpreted to be a normal fault, as the motion of the eastern part of the fault is moving in extension away from the western part (Fig. 1a). This interpretation is in agreement with the inversion of the focal mechanisms of seismic aftershocks in the vicinity of the fault^7^ and differ from the neighboring faults, located 10–20 km to the east, that were interpreted as reverse faults^14,15^.

## Fault segmentation, diffuse deformation and supershear rupture

**Figure 4:**
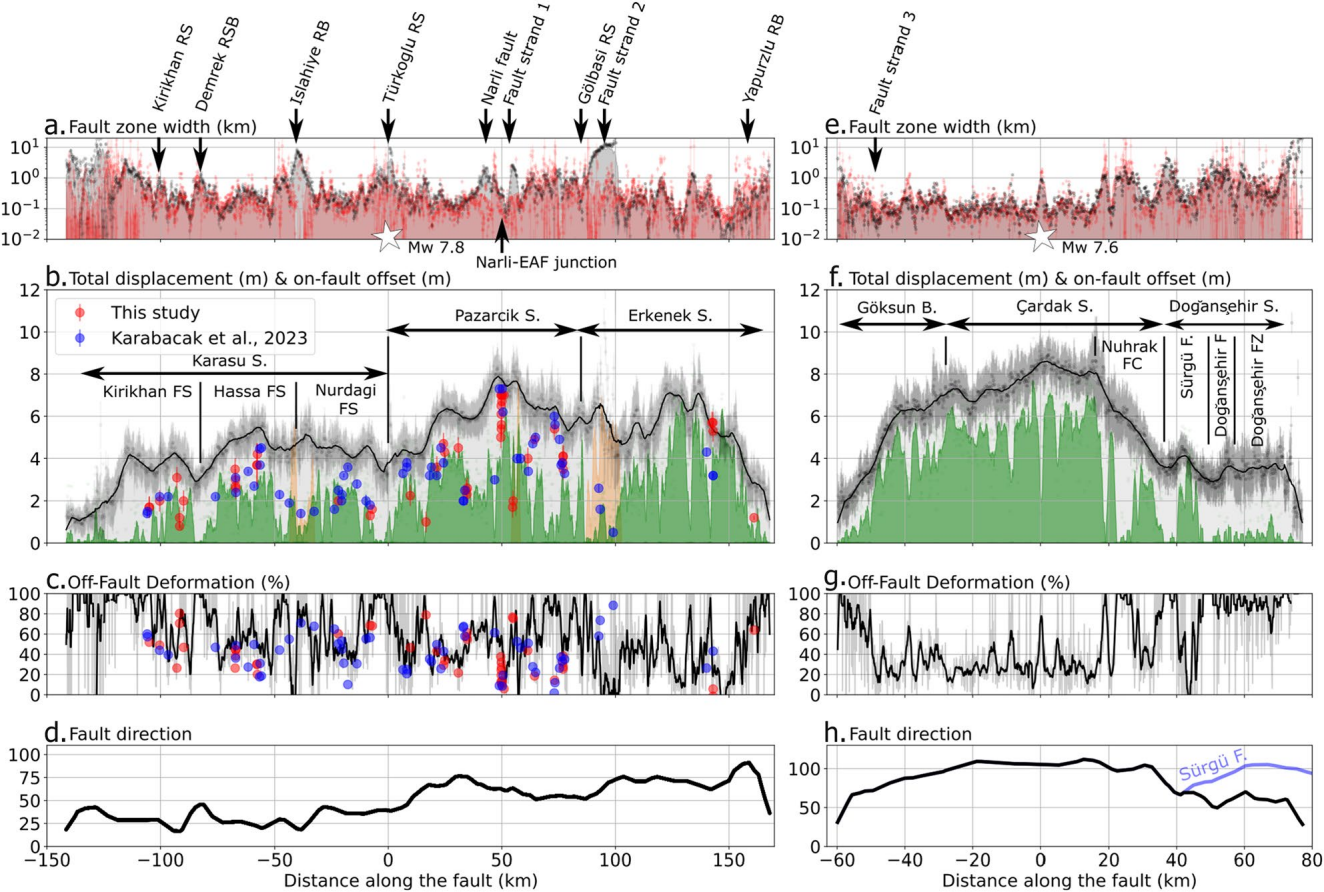
Distribution of fault widths, offsets and diffuse deformation: (**a,e**) show the fault widths computed over 20 km long cross-profiles (black) and 5 km long cross-profiles (red) for the Mw7.8 rupture (left) and Mw7.6 ruptures (right). The location of fault splays and structural complexities are indicated by arrows, and the location of the epicenter is indicated by white stars. (**b,f**) The total offsets ($D_{tot}$) computed on 20 km long profiles (black) using linear regression of far-field displacement and the on-fault offset ($D_{on}$) computed on 5 km long profiles (green) using the gradient criteria to determine the fault location (see Methods for details). The offsets measured in the field by this study and the one by^6^ are plotted as red and blue dots for the Mw 7.8 rupture (left). The segmentation of each rupture is also indicated. (**c,g**) The fault direction of the fault along the main ruptures. The fault direction of the Sürgü fault is also indicated in blue in (**g**).

The fault traces show a succession of narrow segments and diffuse zones or branches that can be mapped with high accuracy and many details (Fig. 3). The 10 m spatial resolution of the Sentinel-2 images also allows estimating the fault width and offset to be accurately estimated (Figs. 2d–g,  4). For the Mw 7.8 rupture, the mean fault zone width is 1 km with a standard deviation of 2.5 km (1$\sigma$) when considering fault splays (Fig. 3a), and 466 m (1.5 km, 1$\sigma$) when considering only the main rupture trace (Fig. 4a). The width of the surface rupture is heterogeneous along the entire rupture length, with a succession of wide (> 500 m) and narrow (< 150 m) ruptures (Fig. 4a). The main complexities of the Demrek restraining bend, the Islahiye releasing bend, the Türkoğlu releasing stepover, and the Gölbaşı releasing stepover described in^14^ are well identified and correspond to wider rupture zones (Fig. 4a). The total offset shows an asymmetric distribution with larger offsets (i.e. 5 to 7 m) in the northeastern part of the rupture (with respect to the epicenter) and smaller offsets (< 5.5 m) towards the south. The mean total offset is of 4.8 m ± 1.6 m (1$\sigma$) and reaches a maximum of 7.5 m ± 0.8 m where we locate the junction of the Narlı fault and the EAF (Fig. 4b). This value and its location are in agreement with the measured offset on the field of 7 ± 0.5 m (this study, Fig. 2d, e) and the previous field survey (7.3 ± 0.2 m^6^). Based on the width of the fault zone and on the measured total offset, we can identify three main segments as defined by^14^: the Karasu, Pazarcık and Erkenek segments for the Mw 7.8 rupture (Fig. 4a, b) with an average total offset of 3.8 ± 1.2 m (1$\sigma$), 6.0 ± 1.1 m (1$\sigma$), 5.3 ± 1.5 m (1$\sigma$) and an average fault width of 1.0 ± 2.7 km (1$\sigma$), 0.7 ± 0.9 km (1$\sigma$), 1.3 ± 3.1 km (1$\sigma$), respectively. We observe that the offset progressively decreases between the Pazarcık and Karasu segments, where the rupture direction changes smoothly (Fig. 4d). Considering the on-fault displacement (Fig. 4b), three additional fault sections may be considered within the Karasu segment: the Nurdağı, Hassa and Kırıkhan fault sections. Most of the models have used a delineation of 2 to 3 main segments to model the Mw 7.8 rupture^27,28,42,43^ being consistent with the main orientation of the rupture and the distribution of the total offsets (Figs. 3a,  4b). However, we also note that the southern part of the Karasu segment may be more accurately separated into three sub-segments which is consistent with the model proposed by^7^ and with the sub-events identified by^11^.

The diffuse deformation is computed from the on-fault and total offsets (see Methods for details) and an average diffuse deformation of 58% is found for the Mw 7.8 rupture. It shows high variability along the fault (Fig. 4c), with fault width and diffuse deformation increasing at segment boundaries and at the extremities of the fault. In between the structural complexities, very localized deformation occurs on very narrow fault zones and is prone to have hosted supershear rupture^44,45^. Such zones are observed on very short sections of the main strand of the EAF, such as between the Narli-EAF junction and the Türkoglu releasing stepover, or between the Islahiye releasing bend and the Demrek restraining bend. There is no consensus on whether a supershear rupture occurred on the main strand of the EAF. Some studies argue for a total subshear rupture^11^ while others argue for local supershear segments^29,30,46^. For instance, Wang et al. (2023)^46^ proposed a supershear rupture between the Narli-EAF junction and the Gölbasi releasing stepover (Figs. 3c, 4a). In this part of the EAF, the average width calculated from the satellite displacement field is not particularly narrow (1 ± 2.5 km), suggesting that the rupture is unlikely to be supershear. However, the estimate may be biased in this part as the on-fault offset is significantly different from the field measurement (Fig.  4b), probably due to a noisier correlation (Fig. S7). In addition, in this section of the fault, low aftershock density (Fig. 3a) and the low slope gradient of the topography in this part of the fault favour supershear rupture^44,47^. Supershear rupture is also detected on seismic strong motion observations^30^ around the Demrek restraining bend and along the southern end of the Karasu segment. To the north and south of the Demrek RSB, we identify short-length (< 10 km) supershear-prone segments. Conversely, at the end of the Karasu segment, fracture width and diffuse deformation increase, both of which are unfavorable for supershear rupture.

For the Mw 7.6 rupture, the width of the fault is narrower, with a mean of 0.7 km and a standard deviation of 2.8 km (1$\sigma$; Fig.  4e). The offsets are larger than for the Mw 7.8 rupture, with a mean total offset of 5.3 ± 2 m and a maximum of 8.7 ± 0.8 m at the epicenter location (Fig. 4f). Similar to the Mw 7.8 rupture, the offset decreases with the fault direction (Fig. 4h). Fault zone width and offset distributions are homogeneous along distinct fault segments. We retrieve some of the structures described by previous studies^14^ and propose three main segments along which the rupture propagates: the Göksun bend, Çardak segment, Doğansşehir segment (Fig. 4e, f). We include the Nuhrak Fault Complex in the Çardak segment, as the direction of the rupture remain the same until the junction between the Sürgü and the Çardak fault (Fig. 4h). To the east of the Nuhrak Fault Complex, the rupture continues along the Sürgü fault for 11 km, then jump in to the Doğansşehir fault for 13 km, and then propagates on an unprecedented mapped zone south to the Doğansşehir fault zone (Fig. 4e, f). The former has usually been gathered in one main segment in proposed models^11,27–29,48^ except for Barbot et al. 2023^7^ who chose to split this part of the fault in two smaller segments. The magnitude of slip varies significantly in this segment from one model to another, and is often slightly underestimated in comparison with measured total offsets of this study in the Doğansşehir segment^7,27,43,48,49^. He et al. 2023^42^ seems to retrieve magnitude of shallow slip in agreement with our surface observations in this segment, although they do not account for Sentinel-2 displacement fields in their inversion.

The rupture initiates on the Çardak fault with a small stepover near the epicenter location (Fig. 3f) and a short increase in the width of the fault zone (i.e. 1 km). The rupture propagates bilaterally to the east and west along a very narrow surface trace (i.e. 143 ± 138 m (1$\sigma$); Fig. 4e) with large total offsets (i.e. 7.7 ± 0.6 m (1$\sigma$); Fig. 4f) revealing very localized deformation with an average diffuse deformation of only 29%. These observations are very favorable for the development of supershear ruptures^44,47^ and are partially consistent with previous models that retrieved supershear ruptures on the western side of the epicenter^11^. However, we point out that the low slope gradient of the topography and the low number of aftershocks on the eastern side of the epicenter (Fig. 4h) is in favor of a supershear ruptures to the east as well. Moreover, rupture velocity and pre-stress field may also explain why the rupture did not propagate along the entire Sürgü fault but maintained a constant direction towards the Doğansşehir fault zone^50,51^ (Fig. 3f). The diffuse deformation budget is slightly lower for the Mw 7.6 earthquake (47 %; Fig. 4f) than for the Mw 7.8 earthquake (54 %; Fig. 4b) with a remarkably long section of low diffuse deformation (< 30%) on the Çardak segment (Fig. 4f).

## Conclusion

The rupture traces of the 2023 Kahramanmaraş doublet occurred along the known faults of the active faults database^15^ although sometimes slight offsets are observed as well as new fault splay and branches. The main observation is that the rupture does not always propagate along the most expected path, especially in the eastern extremity of the Çardak fault, where the rupture, instead of continuing on the Sürgü fault, propagates along a previously unmapped strand. The currently available models report maximum slip from 8 m^7,11,29,52^ to 10 m^43^ for the Mw 7.8 earthquake and from 6 m^43^ to 12 m^7^ for the Mw 7.6 earthquake. This value would lead to a shallow slip deficit of 7 to 25% and of 0 to 12.5% for, respectively, the Mw 7.8 and Mw 7.6 rupture. These values and the geometrical properties of the fault trace tend to indicate that both faults are mature^53^. Together with accurate rupture trace location and estimation of the near-field offsets that are crucial for rupture velocity and slip modeling^54–56^, these observations are key for future seismic risk assessment in the region.

## Methods

### Sub-pixel image correlation applied to Sentinel-2 optical satellite imagery

We used the GDM-OPT-ETQ processing chain^35^, based on the MicMac image matching library^37^. Sentinel-2 L1C images are processed with a moving window of 5 by 5 pixels, a correlation threshold of 0.8 and a regularization parameter of 0.3^36,37^. By default, two corrections are applied to the displacement fields^57^: (a) a 2D ramp is estimated and removed in order to correct for potential co-registration errors, (b) charge-coupled devices (CCD) shifts are estimated and compensated along the footprint of each Sentinel-2 CCD. No CCD stripes are visible in the final displacement fields (after correction), except for one shift visible in the 37SYF tile, which was manually corrected by removing a constant shift of 1.2 m in the affected stripe (Fig. S8). The displacements are given in the East-West (EW) and North–South (NS) directions and are merged over the entire area at full resolution (i.e. 10 m $\times$ 10 m). The GDM-OPT-ETQ processing chain is deployed on the high performance computing (HPC) cluster (A2S) of the University of Strasbourg. The final products were obtained within 10 hours. The first pair of images was processed on February 9, 2023 and the dataset has been released in open access (10.25577/EWT8-KY06^58^). The co-seismic displacements are estimated between the Sentinel-2 acquisitions of January 25, 2023 and February 9, 2023. The presence of snow in the image of February 9, 2023 introduces artifacts, mainly visible in the North-South component (Fig. S1a, b), leading to a much higher estimate of the co-seismic displacements. To remove the snow artifacts and increase the spatial density of the measurements, a second pair of images is considered with the Sentinel-2 acquisitions of May 25, 2022 and May 5, 2023 (i.e. first cloudless and snow-free acquisition after the earthquakes). The same processing parameters are applied to the two pairs of images. The displacement field for this pair is less noisy (Fig. S1c, d) revealing the full extent of the rupture traces. The two products were then averaged (Fig. 1a, b).

Given the date of the second pair, some of the measured displacements may be related to post-seismic. However, we believe that the results are not significantly affected by post-seismic deformation, mainly because the possible magnitude of the post-seismic slip is in the range of Sentinel-2 offset tracking accuracy and field measurement accuracy. In fact, several studies have found that the postseismic slip is about 10 to 20% of the co-seismic slip (e.g., 2021 Maduo earthquake with 0.3 m postseismic slip after 2 months for about 5 m co-seismic slip^59^; 2001 Kokoxili earthquake with 0.6 m postseismic slip after 6 years for about 9 m co-seismic slip^60^). In the case of the 2023 Kahramanmaraş doublet, Xu et al. 2023^49^ found about 5 cm of postseismic slip between mid-February and mid-April 2023 using the Sentinel-1 interferogram. Extending this value to May 2023 would result in about 7.5 cm of post-seismic deformation. About 2.4 mm/week of deformation has been reported by creepmeters at Goksun, resulting in about 40 cm of post-seismic slip in May 2023. Sentinel-2 offset tracking has an accuracy of about 1 meter (1/10th of a pixel) and it is impossible to distinguish between the inherent measurement error and possible post-seismic slip. Although this error may seem important, we believe that the high density of our measurement points allows us to obtain an accurate estimate of the co-seismic slip distribution.

### Fault trace, fault width and fault offsets

The fault trace is first manually mapped from the interpretation of the co-seismic displacement fields (Fig. 1a, b). The fault direction (azimuth) is then computed every 10 m, allowing the EW and NS displacements to be transformed into parallel and normal to the fault displacement along cross-sections of 20 km centered on the fault trace. This strategy is used because the direction of the fault varies significantly along the two ruptures. To measure fault widths and total fault displacements, the displacement profiles parallel to the fault are averaged over boxes of 100 m width. The fault width is then derived every 50 m by fitting an arctangent function^61^ to the data:

1 $$\begin{aligned} y = -\frac{s}{\pi }\arctan ({\pi \frac{x-x_0}{w_c}}) + k_0 + k_1x \end{aligned}$$

where *s* is the total fault slip, $x_0$ is the rupture location (adjusted by the model if necessary), $w_c$ is the fault width, k0, k1 are constants to correct for far-field displacement and linear trend. This law is used to determine the interseismic locking depth^62^, which approximates the crust as a screw dislocation^61^, and is used here to determine the width of the fault zone (Fig. 2e, g). The total displacement is then determined by estimating the linear trend on either side of the fault zone and extrapolating the displacement at the center of the fault zone (Fig. 2e, g). This allows the near-field deviation from the 2D elastic model^26^ to be accounted for. Second, we derive on-fault displacements from 2.5 km cross sections. The inelastic part of the fault is defined as the portion of the fault zone where the absolute value of the gradient is greater than 0.02 m/m (in absolute value), allowing the on-fault offsets to be determined (Fig. 2e–g). This value was chosen from the analysis of the profiles and is in agreement with a recent threshold determined from the analog fault experiment^63^. A lower threshold of 0.5%^23,24,64^, corresponding to the rock yield strength derived from laboratory experiments, is commonly used, although this value may not be valid for all rock types, fault geometries, or rupture processes^34^.

Besides satellite imagery, a field campaign was conducted in May 2023 over the Mw7.8 rupture to collect in-situ fault offsets at 48 locations (Fig. 1b). The measured offsets range from 0.7 to 7 m (Fig. 1c). The offsets were measured along the fault directions by several people in the team. This allowed to determine errors on most location points, ranging from 10 cm to 1 m.

### Supplementary Information

Supplementary Information.

## Data Availability

The Sentinel-2 scenes are available from the Copernicus Open Access Hub at https://scihub.copernicus.eu/. The GDM-OPT-ETQ service is developed and maintained by Data-Terra/ForM@Ter (Data and Service Hub for the Solid Earth: poleterresolide.fr) and exploited on the EOST/A2S High Performance Computing (HPC) infrastructure of University of Strasbourg (1.5 Tier Mesocentre). The service is accessible on-demand through the ForM@Ter webservice (en.poleterresolide.fr/260services-en/gdm-en/#/optic) and/or through the Geohazards Exploitation Platform (GEP: geohazards-tep.eu). The datasets generated during the current study are available in a Form@ter repository with an CC-BY-NC license under https://doi.org/10.25577/EWT8-KY06 for the displacement maps and 10.57932/dc1c9cd3-0e7b-4b25-8344-9dc1c8f14c49 for the rupture traces, offsets and widths.
