# Supplementary material for: High-resolution co-seismic fault offsets of the 2023 Türkiye earthquake ruptures using satellite imagery
Source: Sci Rep. 2024 Mar 21;14:6834. doi: 10.1038/s41598-024-55009-5 (PMC10957881; doi:10.1038/s41598-024-55009-5)
Supplement: Supplementary file 1 — Supplementary Information. [file 41598_2024_55009_MOESM1_ESM.pdf]

Supplementary Information for

Supplementary Information for : High-resolution co-seismic fault offsets of the 2023 Türkiye earthquake ruptures using satellite imagery

Floriane Provost[1,2], Volkan Karabacak[3], Jean-Philippe Malet[1,2], Jérôme Van der Woerd[1,2], Mustapha Meghraoui[1,2], Frédéric Masson[1,2], Matthieu Ferry[4], David Michéa[1], Elisabeth Pointal[5]

1. Ecole et Observatoire des Sciences de la Terre (EOST), CNRS UAR 830 - Université de Strasbourg, 5 rue René Descartes, F-67084 Strasbourg, France
2. Institut Terre et Environnement de Strasbourg (ITES), CNRS UMR 7063 - Université de Strasbourg, 5 rue René Descartes, F-67084 Strasbourg, France
3. Department of Geological Engineering, Eskisehir Osmangazi University, 26040 Eskisehir, Türkiye
4. Géosciences Montpellier, Université de Montpellier - CNRS, Montpellier, France
5. Data-Terra / Pôle de Données Terre Solide (ForM@Ter), CNRS - Institut de Physique du Globe de Paris, 1 rue Jussieu, F-75005 Paris, France

Content of this file

- **Figure S1**
- **Table S1**
- **Figure S2**
- **Figure S3**
- **Figure S4**
- **Figure S5**
- **Figure S6**
- **Figure S7**

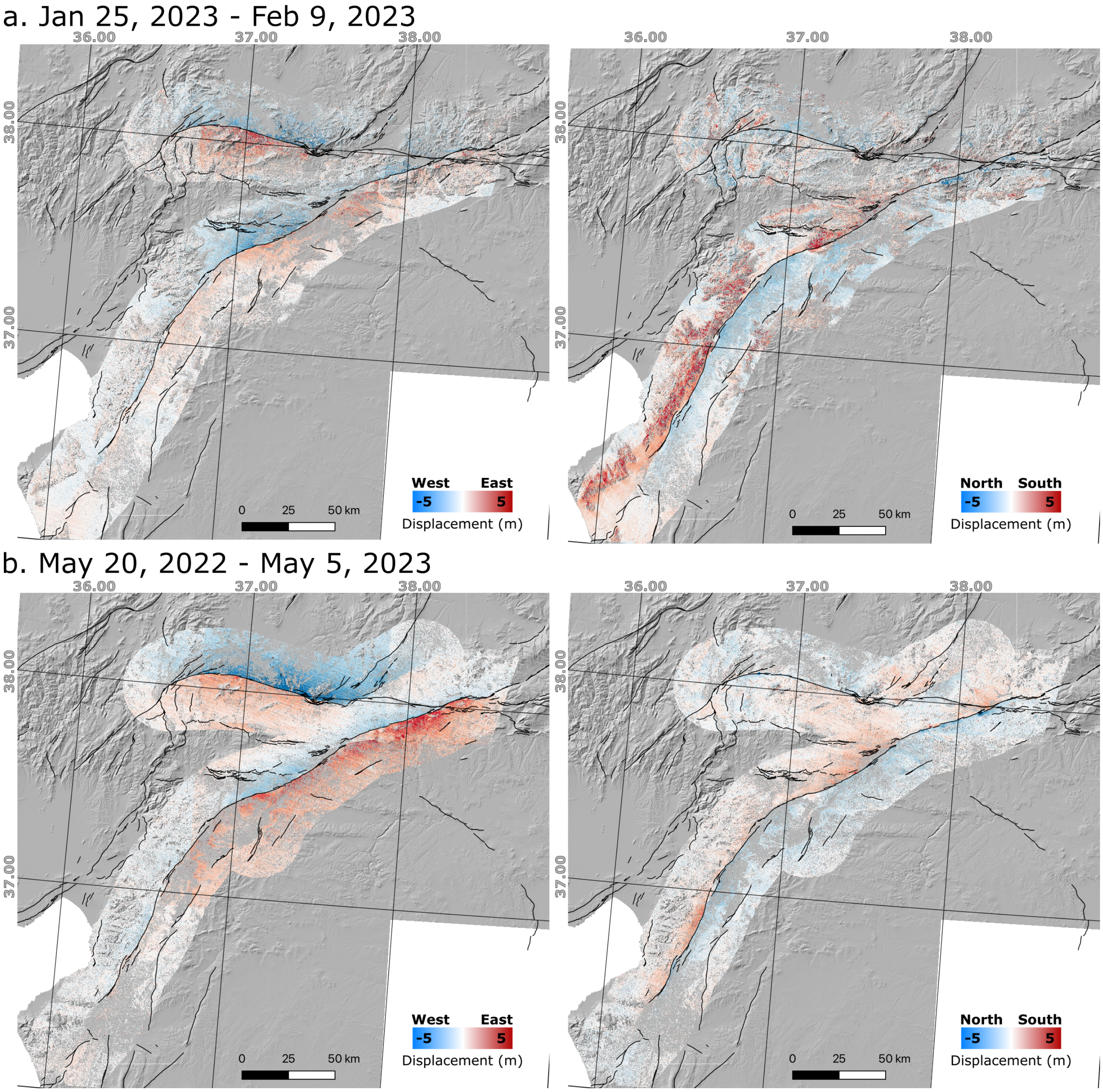

Figure S1: East-West and North-South displacement fields for two pairs of Sentinel-2 images. a) Pair between January 25 and February 9, 2023; b) pair between May 20, 2023 and May 5, 2023.

Pair 1	
S2A_MSIL1C_20230209T082111_N0509_R121_T36SYF_20230209T091429.SAFE	S2B_MSIL1C_20230125T082129_N0509_R121_T36SYF_20230125T090640.SAFE
S2A_MSIL1C_20230209T082111_N0509_R121_T36SYG_20230209T091429.SAFE	S2B_MSIL1C_20230125T082129_N0509_R121_T36SYG_20230125T090640.SAFE
S2A_MSIL1C_20230209T082111_N0509_R121_T37SBA_20230209T091429.SAFE	S2B_MSIL1C_20230125T082129_N0509_R121_T37SBA_20230125T090640.SAFE
S2A_MSIL1C_20230209T082111_N0509_R121_T37SBB_20230209T091429.SAFE	S2B_MSIL1C_20230125T082129_N0509_R121_T37SBB_20230125T090640.SAFE
S2A_MSIL1C_20230209T082111_N0509_R121_T37SBC_20230209T091429.SAFE	S2B_MSIL1C_20230125T082129_N0509_R121_T37SBC_20230125T090640.SAFE
S2A_MSIL1C_20230209T082111_N0509_R121_T37SBV_20230209T091429.SAFE	S2B_MSIL1C_20230125T082129_N0509_R121_T37SBV_20230125T090640
S2A_MSIL1C_20230209T082111_N0509_R121_T37SCB_20230209T091429.SAFE	S2B_MSIL1C_20230125T082129_N0509_R121_T37SCB_20230125T090640.SAFE
S2A_MSIL1C_20230209T082111_N0509_R121_T37SCC_20230209T091429.SAFE	S2B_MSIL1C_20230125T082129_N0509_R121_T37SCC_20230125T090640.SAFE
S2A_MSIL1C_20230209T082111_N0509_R121_T37SDB_20230209T091429.SAFE	S2B_MSIL1C_20230125T082129_N0509_R121_T37SDB_20230125T090640.SAFE
S2A_MSIL1C_20230209T082111_N0509_R121_T37SDC_20230209T091429.SAFE	S2B_MSIL1C_20230125T082129_N0509_R121_T37SDC_20230125T090640.SAFE
**Pair 2**	
S2B_MSIL1C_20220520T081559_N0400_R121_T37SBC_20220520T091256	S2B_MSIL1C_20230505T081609_N0509_R121_T37SBC_20230505T085532
S2B_MSIL1C_20220520T081559_N0400_R121_T37SBA_20220520T091256	S2B_MSIL1C_20230505T081609_N0509_R121_T37SBA_20230505T085532
S2B_MSIL1C_20220520T081559_N0400_R121_T37SDC_20220520T091256	S2B_MSIL1C_20230505T081609_N0509_R121_T37SDC_20230505T085532
S2B_MSIL1C_20220520T081559_N0400_R121_T37SCC_20220520T091256	S2B_MSIL1C_20230505T081609_N0509_R121_T37SCC_20230505T085532
S2B_MSIL1C_20220520T081559_N0400_R121_T37SCB_20220520T091256	S2B_MSIL1C_20230505T081609_N0509_R121_T37SCB_20230505T085532
S2B_MSIL1C_20220520T081559_N0400_R121_T37SBB_20220520T091256	S2B_MSIL1C_20230505T081609_N0509_R121_T37SBB_20230505T085532
S2B_MSIL1C_20220530T081609_N0400_R121_T37SBC_20220530T091117	S2B_MSIL1C_20230505T081609_N0509_R121_T37SBC_20230505T085532
S2B_MSIL1C_20220520T081559_N0400_R121_T37SDB_20220520T091256	S2B_MSIL1C_20230505T081609_N0509_R121_T37SDB_20230505T085532
S2B_MSIL1C_20230502T080609_N0509_R078_T37SDC_20230502T090413	S2B_MSIL1C_20220517T080609_N0400_R078_T37SDC_20220517T105318

Table S1: List of Sentinel-2 images used to compute the co-seismic displacement fields.

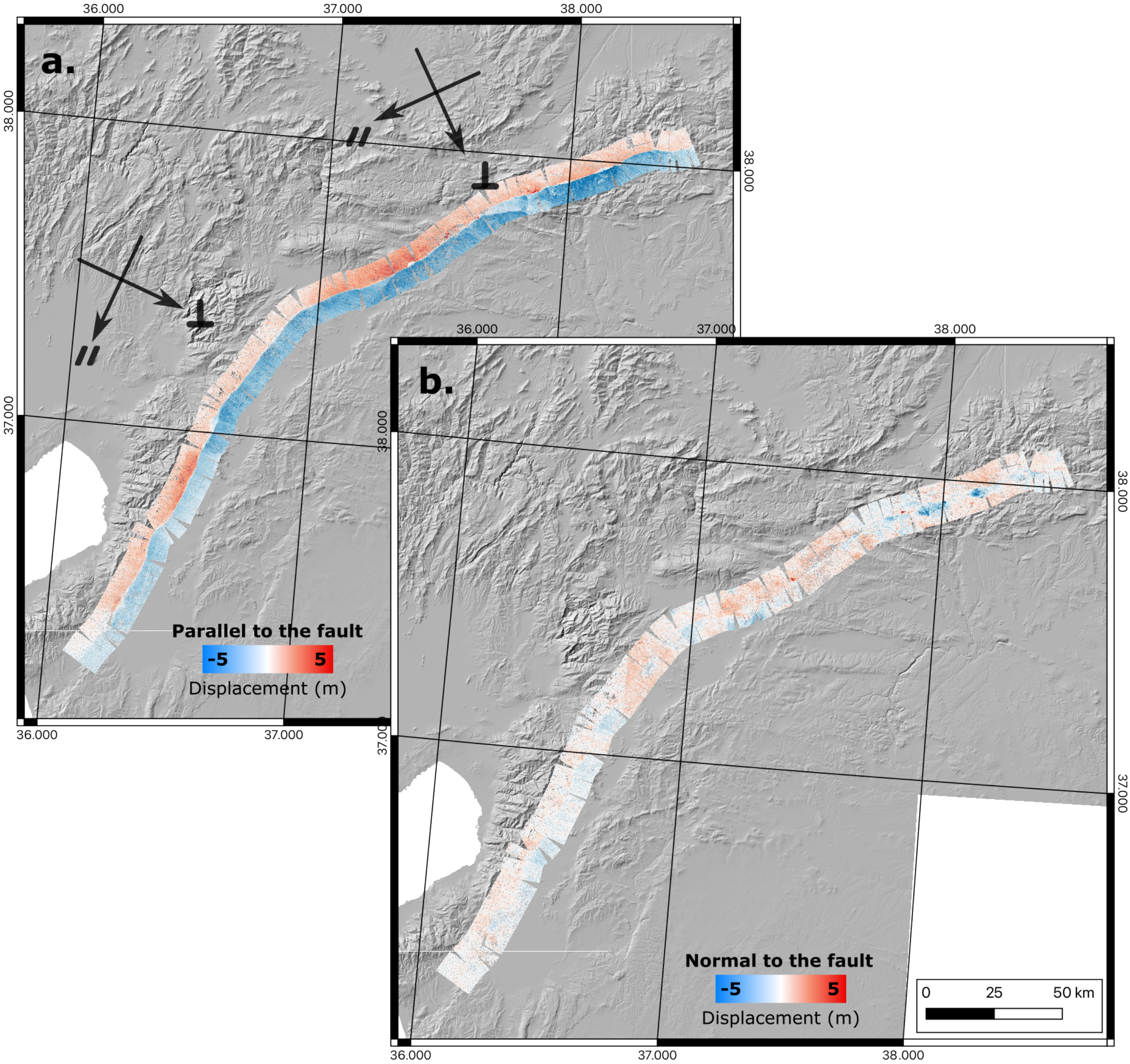

Figure S2: Parallel (a) and normal (b) to the fault displacement for the Mw 7.8 rupture. The EW and NS displacement are rotated along the local direction of the rupture.

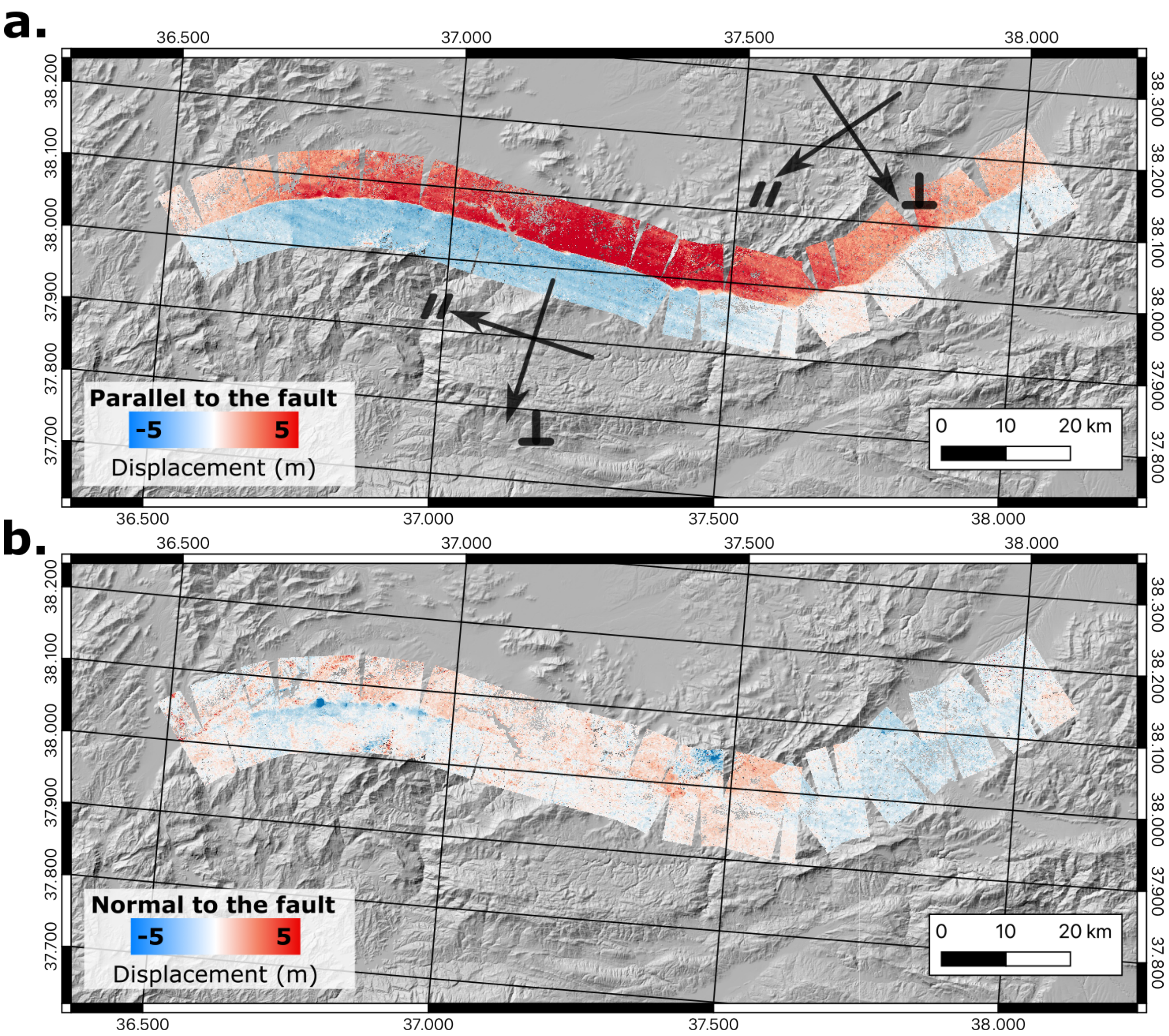

Figure S3: Parallel (a) and normal (b) to the fault displacement for the Mw 7.6 rupture. The EW and NS displacement are rotated along the local direction of the rupture.

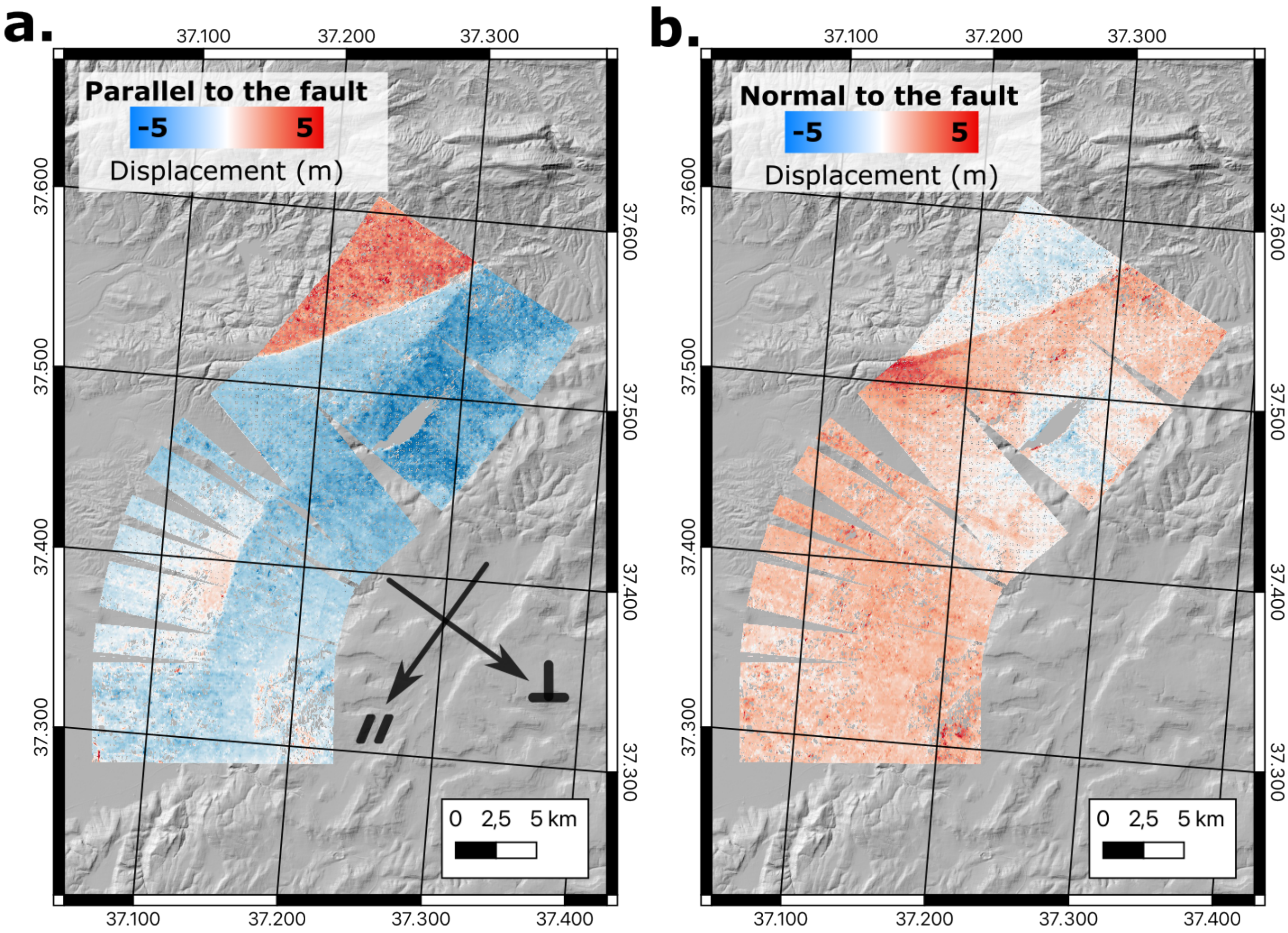

Figure S4: Parallel (a) and normal (b) to the fault displacement for the Narli fault. The EW and NS displacement are rotated along the local direction of the rupture.

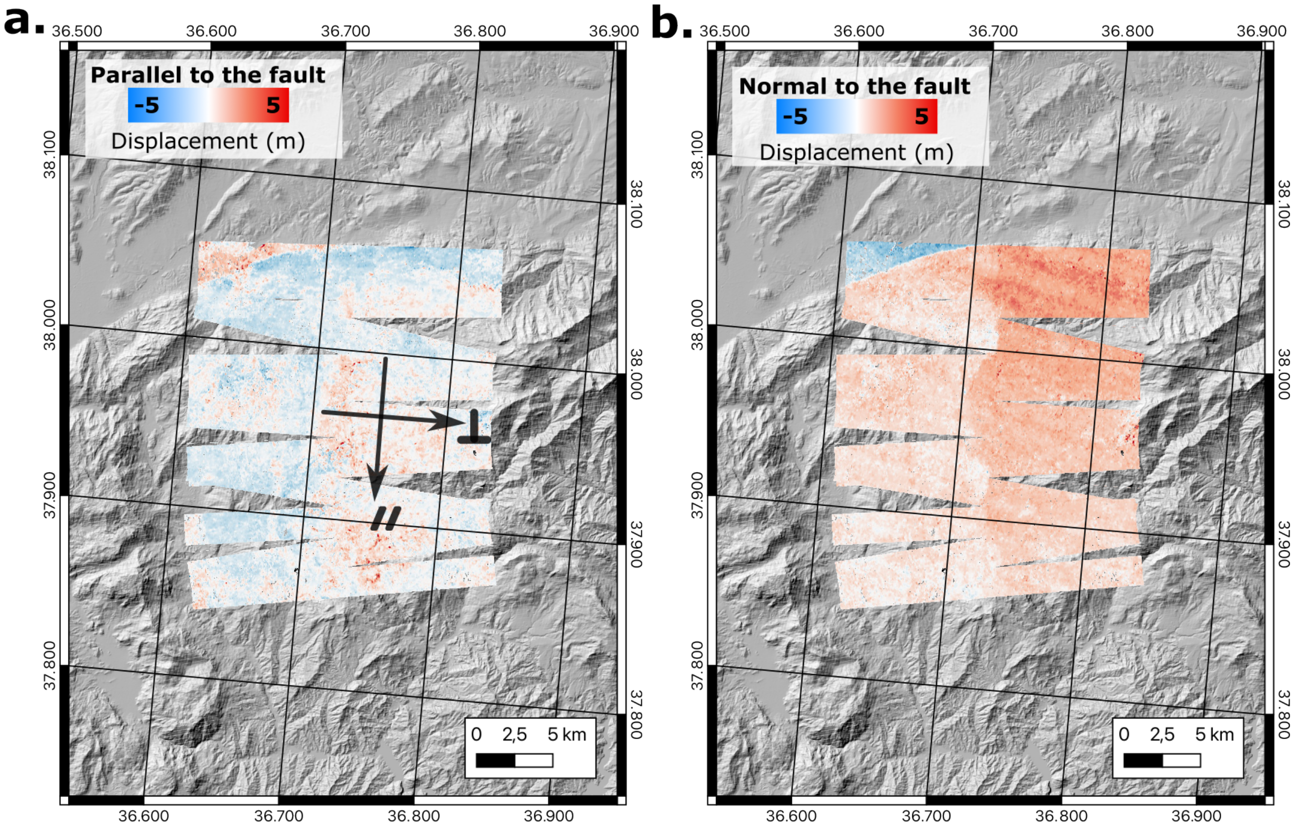

Figure S5: Parallel (a) and normal (b) to the fault displacement for the fault splay 3 located to the south of the Mw 7.6 rupture at the western extremity of Çardak segment. The EW and NS displacement are rotated along the local direction of the rupture.

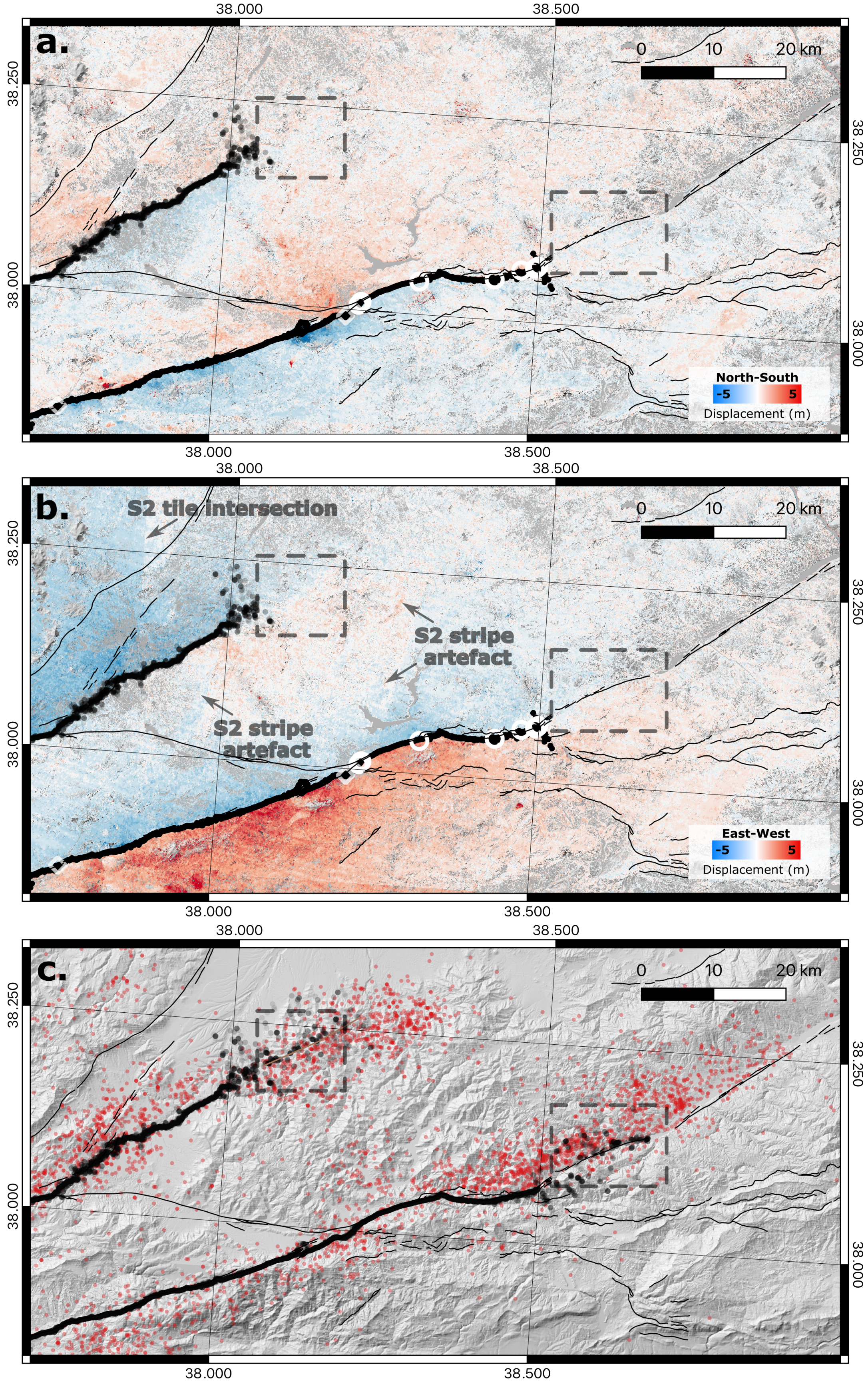

Figure S6: North-South (a) and East-West (b) to the fault displacement for the north-east extremity of Mw7.6 and 7.8 ruptures. The mapped rupture is shown in black dots and active faults are represented in black lines. On the EW displacement, small displacements (< 1.5 m) are visible after at the extremity of the mapped rupture (b) and highlighted by dotted boxes. Figure c) displays the aftershocks (red dots) and the mapped rupture inside the boxes. One can see that the location of the fault is spread suggesting high amount of diffuse deformation.

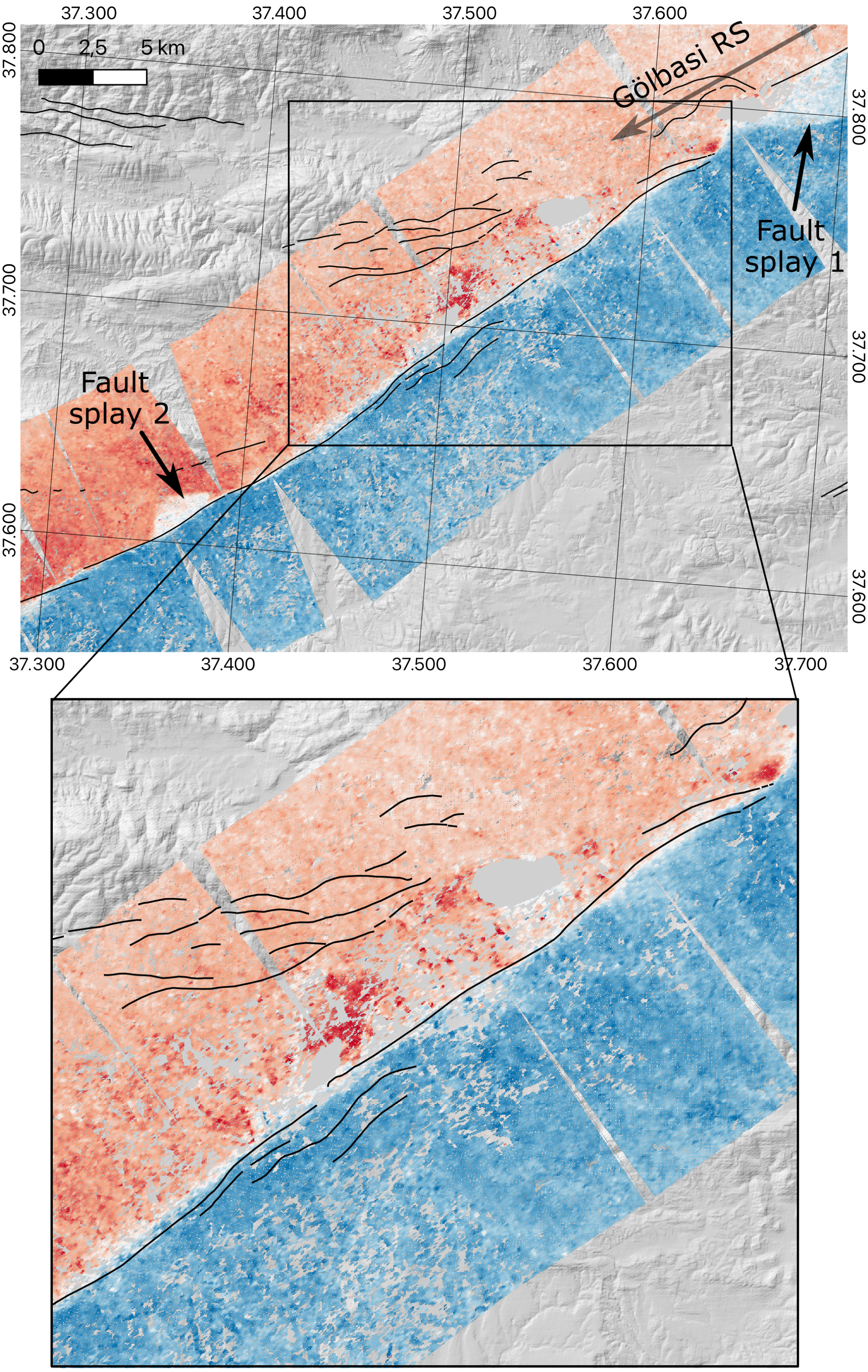

Figure S7: Parallel-to-the fault displacement along the segment between the epicenter of the Mw 7.8 and the Gölbasi RS. The fault trace is fuzzy and very low on-fault offset (< 3 m) are estimated while the total offset are large (> 6 m) and the field offset are estimated to range between 3.5 and 6 m. The displacement fields are less dense than in other part of the fault.

Point	Lat. (°N)	Lon. (°E)	DX (m)	DZ (m)
**1**	37.411765	36.911581	2.25 ± 0.5	0.35
**2**	37.449555	36.976770	1 ± 0.5	
**3**	37.470714	37.025499	3.6	
**4**	38.050398	38.431499	1.20 ± 0.1	1.2 ± 0.2
**5**	38.010341	38.224562	5.3	
**6**	38.010393	38.223997	5.7	
**7**	38.006678	38.220409	5.7	
**8**	37.732466	37.562526	3.5 ± 0.5	
**9**	37.732466	37.562526	4.1	
**10**	37.732230	37.562112	4.1	
**11**	37.731218	37.561181	3.8	
**12**	37.712140	37.528006	5.4	
**13**	37.582654	37.316342	5	
**14**	37.584541	37.320089	7 ± 0.5	
**15**	37.583084	37.316931	7 ± 0.5	
**16**	37.583445	37.317693	6.5 ± 0.1	
**17**	37.583473	37.317736	6.1 ± 0.1	
**18**	37.583827	37.318406	5.4	
**19**	37.584070	37.318921	5.6	
**20**	37.584788	37.320854	6.7 ± 0.1	
**21**	37.585602	37.322474	7 ± 0.1	
**22**	37.589178	37.330279	7	
**23**	37.606802	37.369459	2 ± 0.5	
**24**	37.605668	37.367283	1.7 ± 0.5	
**25**	37.659214	37.453288	4.9 ± 0.2	
**26**	37.639849	37.429144	4	
**27**	37.509503	37.123666	4.5 ± 0.5	
**28**	37.516194	37.166781	2.5	
**29**	37.517228	37.169708	2.3	
**30**	37.517856	37.171278	2.6	
**31**	37.487119	37.059375	4.3	
**32**	37.484987	37.053876	4.7	
**33**	36.801482	36.518879	2.6	
**34**	36.800942	36.518653	2.7	
**35**	36.800403	36.518271	3	
**36**	36.798967	36.517797	3.5	
**37**	36.879662	36.554935	3.7 ± 1	
**38**	36.880732	36.555115	3.7 ± 1	
**39**	36.881814	36.555309	4.2 ± 0.5	
**40**	37.176269	36.713901	2 ± 0.2	
**41**	37.283635	36.795655	1.3 ± 0.2	0.8 ± 0.2
**42**	37.290119	36.801536	1.6 ± 0.2	
**43**	36.624861	36.393111	2 ± 0.5	
**44**	36.608470	36.390445	0.8 ± 0.1	
**45**	36.609056	36.388931	1.2	
**46**	36.597031	36.388889	3.1	
**47**	36.528565	36.370842	2	
**48**	36.493700	36.344132	1.7	

Table S2: Field offset measures along the Mw 7.8 rupture observed in May 2023. Longitude and latitude of measurement points are indicated together with horizontal offset (DX), vertical offset (DZ) when available, and error when estimated.

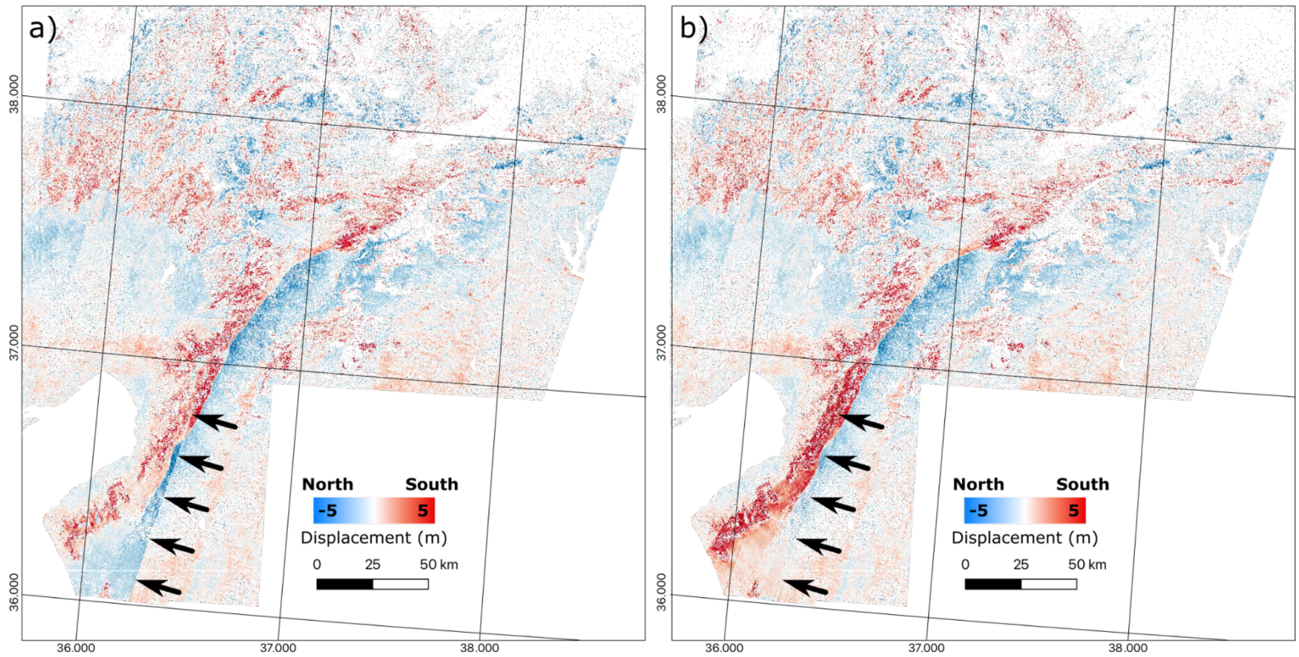

Figure S8: a) Raw North-South displacement field from GDM-OPT-ETQ and b) after manual correction of 1.2 meter on one CCD stripe (black arrows) of the 37SYF tile.
